# Myosin XI-K is involved in root organogenesis, polar auxin transport, and cell division

**DOI:** 10.1093/jxb/ery112

**Published:** 2018-03-22

**Authors:** Mohamad Abu-Abied, Eduard Belausov, Sapir Hagay, Valera Peremyslov, Valerian Dolja, Einat Sadot

**Affiliations:** 1 The Institute of Plant Sciences, The Volcani Center, ARO, HaMaccabim Road, Rishon LeZion, Israel; 2 Department of Botany and Plant Pathology, Oregon State University, Corvallis, OR, USA

**Keywords:** Arabidopsis, auxin, cell division, microtubules, MyoB receptors, myosin XI, root

## Abstract

The interplay between myosin- and auxin-mediated processes was investigated by following root development in the triple myosin knockout mutant *xi-k xi-1 xi-2* (3KO). It was found that the 3KO plants generated significantly more lateral and adventitious roots than the wild-type plants or the rescued plant line expressing functional myosin XI-K:yellow fluorescent protein (YFP; 3KOR). Using the auxin-dependent reporter DR5:venus, a significant change in the auxin gradient toward the root tip was found in 3KO plants, which correlated with the loss of polar localization of the auxin transporter PIN1 in the stele and with the increased number of stele cells with oblique cell walls. Interestingly, myosin XI-K:YFP was localized to the cell division apparatus in the root and shoot meristems. In anaphase and early telophase, XI-K:YFP was concentrated in the midzone and the forming cell plate. In late telophase, XI-K:YFP formed a ring that overlapped with the growing phragmoplast. Myosin receptors MyoB1 and MyoB2 that are highly expressed throughout the plant were undetectable in dividing cells, suggesting that the myosin function in cell division relies on distinct adaptor proteins. These results suggest that myosin XIs are involved in orchestrating root organogenesis via effects on polar distribution of auxin responses and on cell division.

## Teaser

Involvement of myosin XI-K in co-ordinating root development through regulation of auxin transport and cell division.

## Introduction

The plant-specific class XI myosins are the fastest known motor proteins (Higashi-Fujime *et al.*, 1995; Tominaga *et al.*, 2003; Henn and Sadot, 2014). Due to unique enzymatic properties of myosin XIs, plant cells exhibit vigorous cytoplasmic streaming (Woodhouse and Goldstein, 2013) and high velocities of organelle trafficking, reaching 4–5 µm s^–1^ in flowering plants (Nebenführ *et al.*, 1999; Sparkes *et al.*, 2008; Avisar *et al.*, 2009). The model plant *Arabidopsis thaliana* possesses 13 myosin XI genes, some of which, including myosins XI-K, XI-1, and XI-2, are expressed to high levels throughout the plant (Peremyslov *et al.*, 2011). Studies using dominant negative inhibition of myosin activity showed that myosins XI-K, XI-1, XI-2, XI-C, XI-E, and XI-I are important for the trafficking of Golgi stacks, post-Golgi vesicles, mitochondria, peroxisomes, and endoplasmic reticulum (ER) (Avisar *et al.*, 2008, 2009, 2012; Sparkes *et al.*, 2008, 2009). Investigation of single, double, triple, and quadruple myosin knockout plants demonstrated that myosin XI-K was a principal driver of cytoplasmic streaming and organelle trafficking, with myosins XI-1 and XI-2 also contributing to these processes (Peremyslov *et al.*, 2008, 2010; Prokhnevsky *et al.*, 2008; Ueda *et al.*, 2010). Furthermore, it was demonstrated that the velocity of cytoplasmic streaming was determined by myosin motor activity (Tominaga *et al.*, 2013).

It was also found that myosin XI-K and other highly expressed myosin XIs play important roles in polarized growth of root hairs, diffuse expansion of other cell types, as well as in the growth of the aerial plant organs (Ojangu *et al.*, 2007, 2012; Peremyslov *et al.*, 2008, 2010). Strikingly, the contribution of myosins to cell growth was proportional to the cell length, with the longest cells most affected by progressive elimination of myosin XIs (Peremyslov *et al.*, 2010). The velocity of myosin-driven cytoplasmic streaming was identified as a major determinant of myosin function in cell and plant growth (Tominaga *et al.*, 2013). Recently, additional roles of myosin transport in gravitropism, stem positioning, and other aspects of plant morphogenesis were also described (Okamoto *et al.*, 2015; Peremyslov *et al.*, 2015; Talts *et al.*, 2016).

Investigation of the functionally competent myosin XI-K tagged by yellow fluorescent protein (YFP) unexpectedly revealed its association not with the larger organelles such as the Golgi, mitochondria, and peroxisomes, but rather with the vesicle-like compartment that incessantly trafficked along the F-actin bundles (Peremyslov *et al.*, 2012). This vesicular compartment was defined by a novel family of membrane-anchored myosin receptors, MyoBs, that possessed a conserved myosin-binding domain also known as DUF593 (Peremyslov *et al.*, 2013). All six individual MyoB compartments studied so far (MyoB1, MyoB2, MyoB7, MyoB12, MyoB13, and MyoB14) are associated with rapidly moving endomembrane vesicles that are involved in driving cytoplasmic streaming which, in turn, carries passively moving organelles and other vesicular compartments including secretory and endocytic vesicles (Peremyslov *et al.*, 2013, 2015; Kurth *et al.*, 2017). Thus, the myosin-MyoB compartments appear to be specialized in driving cytosol dynamics including endomembrane trafficking (Citovsky and Liu, 2017).

Membrane trafficking is also a key factor in auxin-mediated regulation of plant growth and development in general and polar auxin transport driven by auxin transporters in particular (Adamowski and Friml, 2015). The importance of the ER to Golgi to plasma membrane secretory pathway for polar distribution of auxin transporters was validated using brefeldin A (BFA) that inhibits ADP-ribosylation factor-guanine nucleotide exchange factors (ARF-GEFs), which, in turn, are involved in the post-Golgi pathway and endosome recycling (Satiat-Jeunemaitre *et al.*, 1996; Geldner *et al.*, 2003; Boutté *et al.*, 2006; Naramoto *et al.*, 2014). The ARF-GEF GNOM is a direct target of BFA that partitions to a BFA compartment together with the auxin transporter PIN1 (Geldner *et al.*, 2003). Additional post-Golgi trafficking regulatory proteins involved in the polar distribution of the auxin transporter PIN1 are ARF1A/1C and RabA1b (Feraru *et al.*, 2012; Tanaka *et al.*, 2014). The roles of multiple components and regulators of the endocytic pathways for polar distribution of PIN1 and PIN2 were also firmly established (Teh and Moore, 2007; Tanaka *et al.*, 2009, 2013, 2014; Naramoto *et al.*, 2010; Kitakura *et al.*, 2011; Wang *et al.*, 2013).

In this work, we investigated the possibility that myosin transport contributes to auxin signaling using Arabidopsis root as a sensitive model of auxin-responsive development. Our results imply functional interplay between the myosin- and auxin-dependent processes. Moreover, we showed that myosin XI-K is targeted to the growing cell plate during division of root and shoot meristem cells, probably in a MyoB1- and MyoB2-independent manner, suggesting that myosin function in cell division is distinct from driving cytoplasmic streaming.

## Materials and methods

All materials were purchased from Sigma (Rehovot, Israel) unless otherwise mentioned. The Alexa Fluor^®^ 594-conjugated antibodies were from Jackson ImmunoResearch Laboratories (West Grove, PA, USA).

### Arabidopsis plants, plasmids, and transformation

Arabidopsis seeds were germinated and transformed as previously described (Clough and Bent, 1998). Plasmids containing the DR5_pro_:venus and PIN1prom:PIN1::GFP (green fluorescent protein), which were transformed into *3KO* plants, were kindly provided by the Elliot Meyerowitz (Heisler *et al.*, 2005) and Jiri Friml (Wisniewska *et al.*, 2006) labs, respectively. At least three independent homozygous transformed lines were examined for each transformation. Additional plant lines included wild-type *Arabidopsis thaliana* ecotype Columbia, as well as previously described lines *xi-k xi-1 xi-2* (3KO) and *xi-k xi-1 xi-2 XI-K:YFP* (3KOR) (Peremyslov *et al.*, 2008, 2010, 2012), *xi-k XI-K:mCherry* (Peremyslov *et al.*, 2013), *myob1 MyoB1-YFP*, and *myob2 MyoB2-YFP* (Peremyslov *et al.*, 2013; Kurth *et al.*, 2017). In each of the latter four lines, the genomic clones of Arabidopsis genes under control of the native promoters were used to introduce the fluorescent protein ORF upstream of the termination codon of the corresponding myosin XI-K or MyoB ORFs. The functionality of the resulting tagged proteins was validated for myosin XI-K:YFP (Peremyslov *et al.*, 2012), as well as for MyoB1–YFP and MyoB2–YFP (Peremyslov *et al.*, 2015). The DR5_pro_:venus seeds (Laskowski *et al.*, 2008) and PIN1prom:PIN1::GFP (Blilou *et al.*, 2005) seeds were kindly provided by Ben Scheres. The mCherry–MBD microtubule (MT) marker was a gift from Sabine Müller (Lipka *et al.*, 2014). Two independent lines of each 3KO-mCherry MBD and 3KOR-mCherry MBD were examined.

### Induction of lateral and adventitious root formation

The adventitious roots (ARs) were induced in intact plants as previously described (Gutierrez *et al.*, 2009; Abu-Abied *et al.*, 2012; Rasmussen *et al.*, 2012). Briefly, seeds were germinated on Murashige and Skoog (MS)/0.8% agar plates supplemented with 3% sucrose. The plates were kept in the dark for 2 d at 4 °C, then placed vertically for 5 d at 22 °C in the dark, 4 d in the light (16 h light/8 h dark cycle) when lateral roots (LRs) were counted, and 9 d in the light when ARs from hypocotyls were counted using a stereoscope. Sensitivity to auxin was determined by following root elongation on vertical plates. The 4-day-old seedlings were transferred to MS plates containing 0.05 µM or 0.5 µM indole-3-acetic acid (IAA) and the root length was measured after 5 d; the number of LRs in each root and the root density were calculated. Each treatment experiment included 10–15 plants and was reproduced three times.

### Immunostaining

Immunostaining was performed as previously described (Chaimovitsh *et al.*, 2012) with modifications. The 1-week-old seedlings were fixed for 1 h at room temperature in freshly prepared 8% paraformaldehyde in PME buffer containing 100 mM PIPES pH 6.89, 5 mM MgSO_4_·7H_2_O, 5 mM EGTA, 1% Triton X-100, 1 mM phenylmethylsulfonyl fluoride (PMSF). Samples were rinsed three times (15 min) in PME buffer containing 1% Triton X-100 and treated with the following enzyme mixture: 2% cellulase ‘Onzuka’ R-10 and 1% pectinase in PME buffer containing 1% Triton X-100 and 1 mM PMSF for 10 min. After washing in PME buffer for 3 × 10 min, the samples were put on a charged microscope slide and squashed using a metal block. The squash step was followed by incubation in phosphate-buffered saline (PBS) with 1% Triton X-100 and 10% DMSO for 30 min and then two quick washes with PBS followed by incubation in PBS containing 3% BSA for 1 h. The PIN1- or MT-specific antibodies were diluted 1:500 and 1:100, respectively, in PBS with 3% BSA and incubated with the samples overnight at room temperature in a wet chamber. This step was followed by three washes with PBS for 15 min and incubation with goat anti-rabbit or anti-mouse (1:100) secondary antibodies conjugated to Alexa Fluor 594 (Jackson ImmunoResearch Laboratories, West Grove, PA, USA) for 1 h at room temperature. After three washes with PBS, mounting was done in 50% glycerol/H_2_O and slides were examined with a Leica SP8 confocal laser-scanning microscope. Staining of PIN1 was performed using an antibody kindly provided by Jiri Friml. MTs were stained with DM1A antibody from Sigma. Staining with 1 µM DAPI was done for 15 min during the second wash.

### Microscopy and image analysis

Imaging was done using an SP8 Leica confocal microscope including solid-state lasers emitting 405, 488, 514, and 552nm light, and hybrid or PMT detectors. Objectives were either PL APO ×20/0.75, WD 0.62 mm or PL APO ×63/1.2, WD 0.3 mm. In each experiment, image acquisition parameters were kept similar with no background subtraction. For fluorescence measurements of nuclei, the Imaris (Bitplane A.G.) spot detection option was used to segment nuclei and calculate the average signal intensity. The spectrum-coded color of spots represents the mean fluorescence intensity of the nuclei. Fluorescence of myosin XI-K:YFP and mCherry MTs in dividing cells was acquired every 2 min using line average (×3) and frame accumulation (×2) options. Other quantifications were performed manually. The oblique cell walls were defined by the angles substantially different from 90° or 180° (>110°; <160°) relative to the root axis. Cells exhibiting measurably stronger fluorescence in the basal membrane in relation to the upper and both side membranes were considered to exhibit polarized PIN1 localization.

Measurement of PIN1 fluorescence in basal and longitudinal membranes was done using the line profile tool of Leica Application Suite X software. Cell division orientations, either aligned or tilted with respect to the root axis, were counted in 20 roots of Columbia, 3KO, or 3KOR lines stably expressing the mCherry–MBD marker. Statistical analysis has been performed using Scheffe’s multiple comparison test that was employed due to the uneven number of replicates between the treatments. Statistically significant differences between the treatments were determined using a *P*-value <0.05.

## Results

### Inactivation of the three myosin XIs increases production of the lateral and adventitious roots

**Fig. 1.**
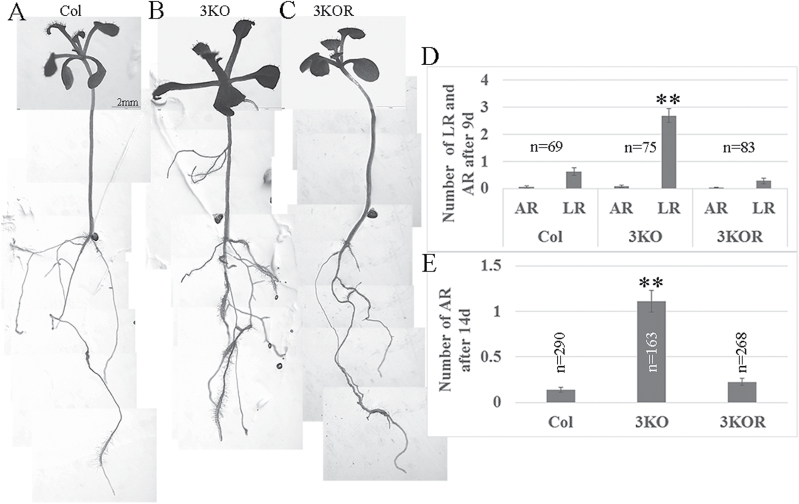
3KO plants generate more lateral (LR) and adventitious (AR) roots than Columbia or 3KOR plants. Seeds were germinated in vertical plates, transferred after 2 d at 4 °C to the growth room, and covered with aluminum foil for an additional 5 d. Four days after the aluminum foil was removed (9 d in total), LRs were counted using a streoscope. For AR counting, plants were grown for 9 d after the dark period (14 d in total). (A–C) Representative plant images after 14 d. (D, E) LRs and ARs were counted after 9 d and 14 d, respectively. Error bars show the SEs; *n*, number of plants for each line. The statistical evaluation was done using Scheffe analysis that implied the significant difference between 3KO plants on one hand, and Columbia or 3KOR plants on the other (marked by double asterisks; *P*<0.01).

ARs are defined as roots that emerge from non-root tissues, in contrast to LRs that develop from roots. Although the exact mechanisms regulating AR and LR formation are distinct (Verstraeten *et al.*, 2014), auxin plays a central role in both. To assess the potential role of highly expressed myosin XIs in the auxin-regulated formation of LRs and ARs, we conducted phenotypic analysis of the triple myosin gene knockout mutant *xi-k xi-1 xi-2* plants (3KO) whose defects in the growth of aerial organs, but not roots, were analyzed previously (Peremyslov *et al.*, 2010). The roots of the 3KO plants were compared with the Columbia control and plants in which the growth phenotype was rescued by the stable expression of the functional myosin XI-K fused to YFP under control of the native *XI-K* promoter (XI-K:YFP; this plant line was designated 3KOR) (Peremyslov *et al.*, 2012). As shown in Fig. 1D, and in Supplementary S1A–C at *JXB* online, after 5 d of plant growth in the dark and 4 d in the light (9 d in total), 3KO plants generated significantly more LRs than Columbia or 3KOR plants. At this stage, hardly any ARs were observed. However, after a further 5 d in the light (14 d total), it was clear that 3KO plants also generate more ARs, than Columbia or 3KOR controls (Fig. 1A–C, E).

To assess the involvement of auxin regulation in the root development phenotype in the 3KO line, plants were grown in the presence of 0.05 µM or 0.5 µM IAA. As expected, LR density was significantly higher in 3KO plants than in the Columbia control plants, but not more than that in 3KOR plants grown without exogenous IAA (Supplementary Fig. S2). In contrast, no statistically significant difference in LR density was found among three experimental variants when the plants were grown in the presence of either IAA concentration (Supplementary Fig. S2J). The ability of exogenous IAA to reverse excessive LR formation in the 3KO plants implied involvement of auxin signaling in the LR phenotype of the myosin-deficient plants.

### Myosin elimination affects root cell morphology, auxin gradient, and polar localization of PIN1 transporter

**Fig. 2.**
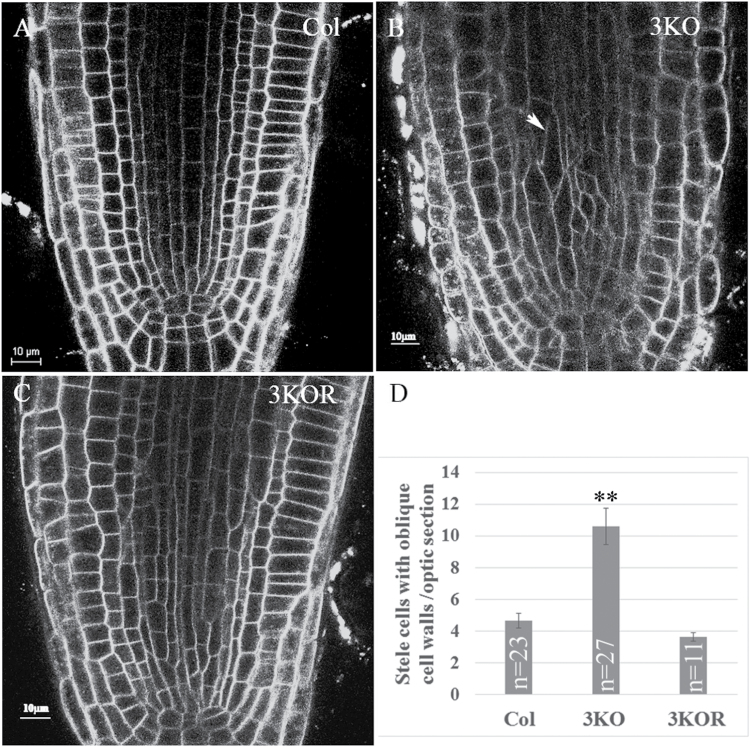
The root tips of the 3KO plants possess more stele cells of irregular shape (including oblique cell walls) and size. Cell membranes were stained with FM4-64. (A, B, C) Columbia, 3KO, and 3KOR plants, respectively. (D) Quantitative analysis of the number of stele cells with oblique walls in the middle optic section of the root tips. Statistical evaluation was done using Scheffe analysis; a significant difference was found for 3KO compared with either Columbia or 3KOR plants (double asterisks; *P*<0.01); *n*, number of roots analyzed for each plant line; bars correspond to the SE.

**Fig. 3.**
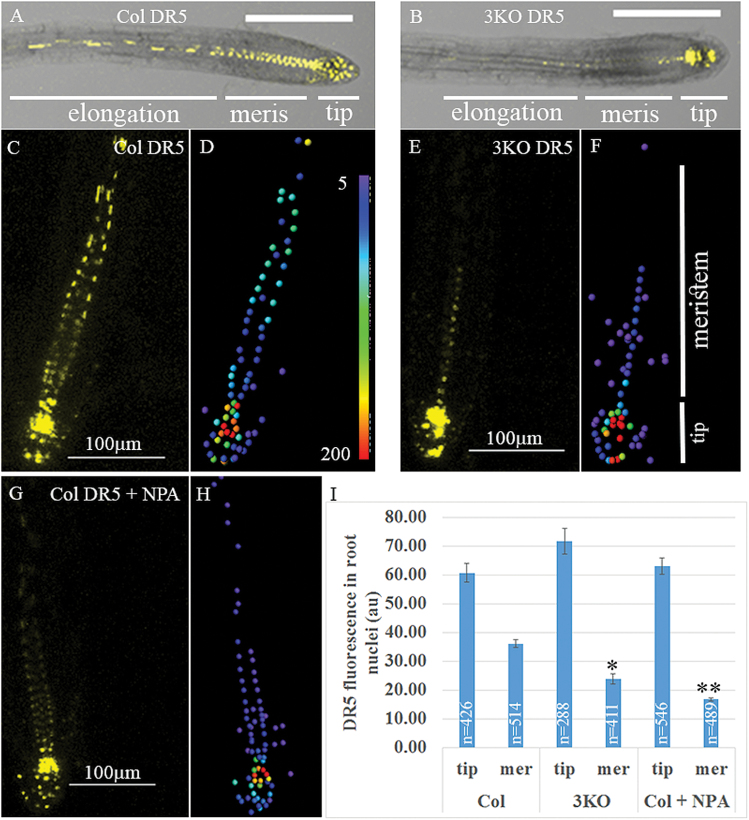
Differential distribution of the nuclear-targeted DR5:venus fluorescence in the roots of Columbia and 3KO plants. Plants were grown for 4 d in the dark and then transferred to plates with MS medium (A–F) or MS medium supplemented with 2 µM NPA (G, H). After 24 h, 10 roots for each treatment were imaged by confocal microscopy; fluorescent nuclei were analyzed by Imaris. (A, B) Representative roots of Columbia and 3KO plants. (C, E, and G) The images of meristematic zones of the analyzed root tips. (D, F, and H) The color-coded fluorescence analysis according to the heat map shown in (D). (I) Quantitative analysis of the fluorescent nuclei in the tip versus the meristematic zone as illustrated in (F). Evaluation of the data by Scheffe analysis showed significant differences between the meristematic zones of 3KO and NPA-treated Columbia roots compared with untreated Columbia. Single and double asterisks correspond to *P*<0.05 and *P*<0.01, respectively. *n*, number of analyzed nuclei. Scale bars are 200 µm in (A) and (B), and 100 µm in (C), (E), and (G). au, arbitrary units.

To determine if myosin elimination also affected the cells in the primary root, we stained the roots with the membrane dye FM4-64 and examined its optical cross-section using confocal laser scanning microscopy. As shown in Fig. 2, although the overall shape of the root tip appeared normal in 3KO plants, the sizes and shapes of many stele cells were irregular and distinct from those in the Columbia and 3KOR roots. In particular, these 3KO cells exhibited more oblique cell walls (those with angles substantially deviating from 90° or 180° relative to the root axis) in contrast to predominantly vertically oriented, elongated, rectangular stele cells in the control plants (Fig. 2). Because stele cells play an important role in basipetal auxin transport to the root tip, we examined potential changes of auxin gradient in 3KO plants using the stably expressed, nuclear-localized, fluorescent DR5:venus marker whose expression level is proportional to the intracellular auxin concentration (Laskowski *et al.*, 2008). As illustrated in Fig. 3A–F and I, the overall distribution of the fluorescent nuclei and corresponding levels of fluorescence were similar in the distal end of root tips of the Columbia and 3KO plants. In sharp contrast, the meristem of the 3KO roots exhibited a significantly reduced level of fluorescence, thus exhibiting a much shorter and steeper basipetal gradient of nuclear fluorescence.

Using the auxin transport inhibitor *N*-1-naphthylphthalamic acid (NPA; 2 µM for 24 h), we observed changes in the auxin gradient that were very similar to those in 3KO plants. More specifically, whereas the ratio of fluorescence between the tip and the above region in Columbia roots was 2:1, it increased to 3:1 in the 3KO plants and NPA-treated Columbia plants (Fig. 3I). Because auxin accumulation at the pericycle and the gradient formed at the primordium are critical for LR formation, we set out to evaluate the DR5:venus signal in the pericycle during early stages of LR formation. Seedlings were examined at 3, 4, and 5 d post-germination and, as shown in Supplementary Fig. S3, accumulation of auxin response signals in 3KO plants was observed at 3 d post-germination preceding that in Columbia plants. Thus, no auxin transport aberrations were detected at this LR developmental stage. Although no direct auxin transport measurements were performed, these data point to an abnormal auxin gradient in 3KO plants, probably due to defects in polar auxin signal distribution in the primary root.

**Fig. 4.**
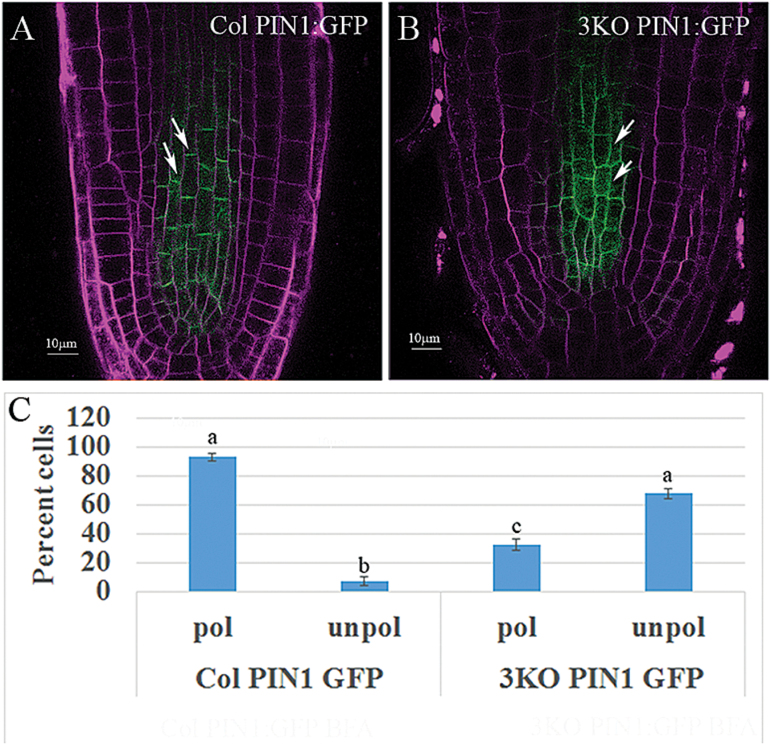
Loss of polar localization of PIN1:GFP in the stele cells of 3KO plants. PIN1:GFP was expressed in 3KO plants and its distribution in stele cells was compared with Columbia plants also expressing this reporter. (A) Columbia PIN1:GFP root tip; arrows mark two cells with typical polarized PIN1:GFP localization. (B) 3KO PIN1:GFP; arrows point to cells exemplifying depolarized PIN1:GFP localization. (C) Quantitative analysis of the proportion of cells (%) showing depolarized PIN1:GFP, evaluated by Scheffe analysis. Letters denote samples showing statistically insignificant (two ‘a’s) and statistically significant (‘a’ versus ‘b’ and ‘c’; ‘b’ versus ‘c’; *P*<0.05) differences. Scale bars=10 µm.

To test the latter possibility, we examined the distribution of the efflux transporter of auxin, PIN1, in the stele cells of Columbia and 3KO plants using PIN1:GFP stably expressed from the native PIN1 promoter (Wisniewska *et al.*, 2006). It was found that 68% of the stele cells exhibited non-polar PIN1:GFP distribution in 3KO plants compared with only 7% in Columbia plants (Fig. 4). Measurements of the mean ratio of PIN1:GFP fluorescence in the basal versus longitudinal membranes yielded values of 0.97 ± 0.2 for 3KO plants versus 2.48 ± 0.8 for Columbia plants (*n*=40; *P*<0.001), suggesting that myosin elimination disrupted the mechanism regulating polar localization of PIN1 in stele cells. Similar experiments using immunostaining with PIN1-specific antibody showed that whereas ~90% of the root stele cells of Columbia and 3KOR plants exhibited preferential localization of PIN1 to the basal membrane, only 55% of these cells in 3KO plants did so (Supplementary Fig. S4). To compare further the ability of Columbia and 3KO stele cells to polarize PIN1, 50 µM or 10 µM BFA treatment and washout assays were performed. Supplementary Fig. S5 shows that, as expected, PIN1:GFP relocated to BFA compartments following BFA treatments, as well as being distributed uniformly throughout plasma membranes in both Columbia and 3KO plants. After 60 min of BFA washout, polar PIN1:GFP distribution was restored in the Columbia plants, whereas in the 3KO plants, PIN1:GFP remained distributed in a non-polar manner throughout the treatments. Collectively, these experimental outcomes suggest that elimination of the three myosin XIs in the 3KO mutant affected the dynamics of endomembrane transport in the root stele cells, thus impairing the polar maintenance of PIN1 at the basal plasma membrane, which in turn affected the formation of a proper auxin gradient.

### Myosin XI-K is concentrated in cell plates

**Fig. 5.**
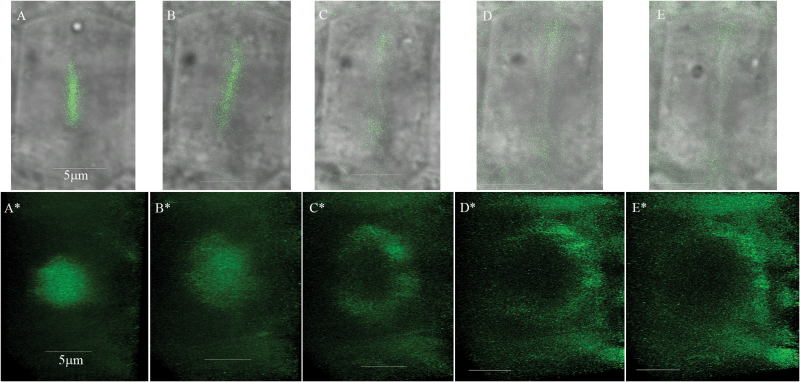
Time-lapse imaging of the myosin XI-K:YFP in 3KOR root meristem cells reveals dynamic disc-like structures that gradually accommodate ring-like shapes. Imaging has been done at 5 min intervals. (A–E) Myosin XI-K:YFP imaging in a single cell. (A*–E*) Corresponding tilted 3-D reconstructions of the myosin XI-K:YFP signal from each time interval performed using a ×63/1.2 objective and *Z* sectioning of 9 µm (total of 61 optic sections, every 0.15 µm with frame accumulation ×3 and resolution 512 × 512). The Leica Hyd detector and speed scan at 600 Hz were used. Scale bars=5 µm.

The irregular morphology of some cell types in multiple myosin XI gene knockout mutants in general (Peremyslov *et al.*, 2010; Ojangu *et al.*, 2012) and oblique cell walls of the 3KO root stele cells in particular (this work) implied a possible role for the myosin XIs in cell wall formation and perhaps in cell division. To address this possibility, we investigated the subcellular localization of XI-K:YFP in the root cells of 3KOR plants that exhibit a virtually normal phenotype due to expression of the functional, fluorophore-tagged myosin XI-K (Peremyslov *et al.*, 2012). Time-lapse imaging of the single cells followed by 3-D reconstructions visualized fluorescent discs that gradually developed into ring-like structures formed by XI-K:YFP, the planes of which were perpendicular to the root axis (Fig. 5).

**Fig. 6.**
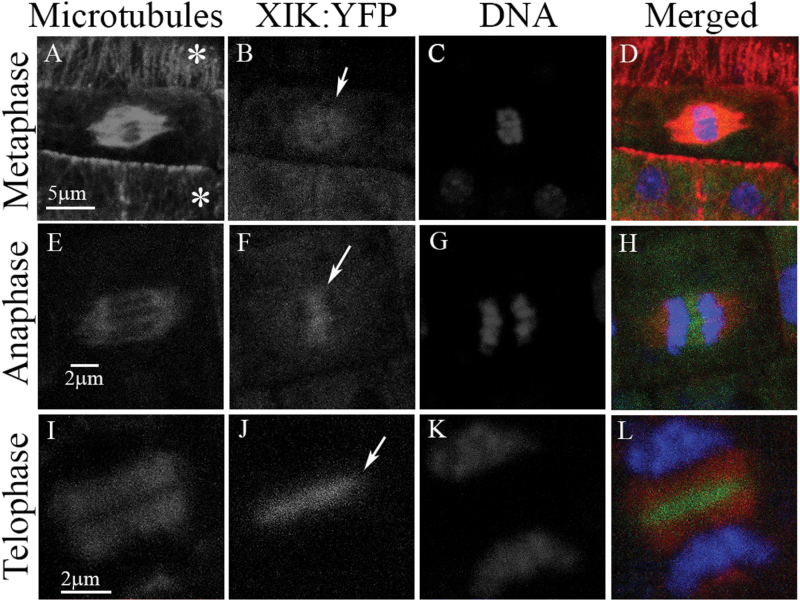
Myosin XI-K:YFP (green) is localized to the cell division apparatus. Microtubules in the root cells of 3KOR plants were immunostained with tubulin-specific antibody (red), and DNA was stained with DAPI (blue). (A–D) Cells in interphase are marked with asterisks. (E–H) and (I–L) Cells in anaphase and telophase, respectively. Arrows in (B), (F), and (J) mark the fluorescent signal of myosin XI-K:YFP at the different cell division stages.

This conspicuous dynamic disc-to-ring-like appearance of myosin XI-K:YFP prompted us to explore a potential connection of this localization pattern to cell division. Strikingly, this connection was validated in an investigation of the root meristematic cells undergoing mitosis using immunochemical detection of MTs. We found that during metaphase, a faint myosin XI-K:YFP signal co-localized with the MTs in the mitotic spindle to the apparent exclusion of centrally positioned chromatids (Fig. 6A–D). During anaphase, myosin XI-K:YFP started to congregate in the mid-zone of the dividing cell, sandwiched between separating chromatids (Fig. 6E–H). Later in telophase, myosin XI-K:YFP was concentrated in the mid-zone of the phragmoplast where the new cell plate is formed (Fig. 6I–L). Therefore, the bright disc seen in Fig. 5 most probably corresponds to myosin XI-K:YFP localized at the emerging cell plate.

**Fig. 7.**
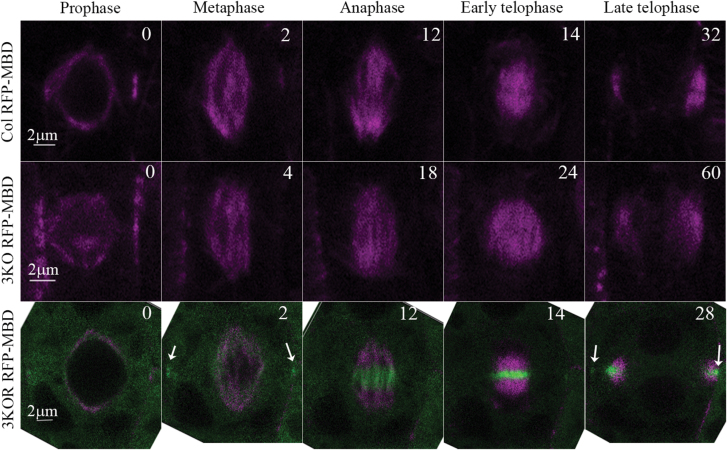
Time course of cell division in Columbia (Col), 3KO, and 3KOR plants. Live imaging of microtubules labeled by the RFP–MBD marker (magenta) and of the myosin XI-K:YFP in 3KOR plants (green) is shown. Numbers in the upper right corners indicate the time (min) from prophase (time 0). Relatively bright myosin XI-K:YFP fluorescence in the cell division zone (CDZ) observed at 2 min and 28 min is marked by white arrows in the bottom row.

To corroborate these data with live cell imaging, we stably expressed the MT marker mCherry–MBD (Lipka *et al.*, 2014) in 3KO and 3KOR plants. By measuring the time from prophase to the end of cytokinesis in each line (*n*=6), it was found that Columbia and 3KOR cells completed the division in 39 ± 7 min and 45 ± 11 min, respectively. In contrast, it took as long as 65 ± 8.5 min for the 3KO cells to complete this process, pointing to a significant difference in the division time (*P*<0.05) due to elimination of the three myosin XIs (Fig. 7). By following the myosin XI-K:YFP signal during prophase, no detectable myosin XI-K:YFP fluorescence overlapping that of MTs was apparent. When the spindle was formed, faint fluorescence of myosin XI-K:YFP overlapped with MTs, whereas brighter fluorescence was observed in the cell division zone (CDZ) marking the future spot of cell plate insertion (Fig. 7, white arrows in the bottom row of the 2 min panel). During anaphase, gradual concentration of myosin XI-K:YFP in the spindle mid-zone was observed. In telophase, myosin XI-K:YFP formed a ring that accompanied MTs in the extended phargmoplast (Fig. 7; Supplementary Movie S1) and exhibited a strong CDZ signal in late telophase (Fig. 7, white arrows in the bottom row of the 28 min panel).

Because the formation and orientation of cell plates are tightly regulated (Rasmussen *et al.*, 2013), the presence of myosin XI-K in the cell division apparatus and cell plates suggested that myosin elimination in the 3KO plants caused aberrations in cytokinesis leading to abnormal stele cell morphologies. Indeed, by following the mCherry–MBD marker it was found that on average, 12.9 ± 4% of dividing cells showed a tilted phragmoplast or spindle, whereas in the Columbia and 3KOR plants, only 2.6 ± 1% and 2.3 ± 1%, respectively, exhibited such tilting (*n*=40 for each line). Representative images of tilted phragmopasts and spindles are shown in Supplementary Fig. S6. To compare the sizes of dividing cells between the Columbia and 3KO plants, roots were stained with DAPI, and the area of one optic section was measured (*n*=15). It was found that these areas were not significantly different between Columbia and 3KO (132 ± 49 µm^2^ and 152 ± 49 µm^2^), suggesting that the tilted spindles were not caused by space constraints in the dividing 3KO root cells. Notably, due to either reduced microscope light penetration or low expression levels in the deeper layers of the root, dividing cells marked by the mCherry–MBD marker were detectable mainly in the root epidermis or cortex, but not in the stele. Therefore, the proportion of abnormally oriented spindles and phragmoplasts in the 3KO plants might be higher in the stele, corresponding to a proportion of oblique cell wall detected in this mutant line (Fig. 2). The relatively low number of tilted dividing cells observed in the outer layers could also explain the apparently normal morphology of the 3KO roots.

It was shown that in the mature cells, most of the myosin XI-K pool is associated with the mobile, vesicle-like compartments defined by highly expressed myosin receptors MyoB1 and MyoB2, along with other less abundant members of the MyoB family (Peremyslov *et al.*, 2013; Kurth *et al.*, 2017). Accordingly, we set out to test whether MyoB1 and MyoB2, each tagged by GFP, were co-localized with myosin XI-K:mCherry (all tagged proteins were expressed under control of their native promoters) in the dividing root cells.

Similarly to myosin XI-K:YFP, myosin XI-K:mCherry was concentrated in the discs or rings in the dividing root tip cells (Supplementary Fig. S7B, H). Surprisingly, no expression of MyoB1–GFP or MyoB2–GFP was detectable in the root tips (Supplementary Fig. S7A, G). In contrast and in agreement with the previous study (Peremyslov *et al.*, 2013), MyoB1–GFP or MyoB2–GFP were concentrated and co-localized with the myosin XI-K:mCherry in the tips of the growing root hairs (Supplementary Fig. S7D–F, J–L). These data imply that the myosin accumulation and potential function within the growing root cell plates involves cargo(s) distinct from MyoB1 and MyoB2 compartments.

To determine if myosin XI-K association with the cell plates is specific to the root tip or is a common property of dividing cells, we imaged the shoot apical meristem in plants stably expressing XI-K:mCherry and MyoB1–GFP. As shown in Supplementary Fig. S8, the pattern of XI-K:mCherry localization in this tissue is very similar to that in the root apical meristem, clearly pointing to myosin’s targeting to growing cell plates. Again, similar to the root tip, no detectable expression of MyoB1–GFP was observed in the shoot apical meristem (Supplementary Fig. S8A). In contrast, MyoB1–GFP was readily observed in the epidermal cells of the growing leaf petioles where, as expected, it was co-localized with myosin XI-K:mCherry (Fig. S8D–F). Thus, concentration of the myosin XI-K, but not its MyoB1 receptor, in the growing plates of dividing cells is a common feature of both the root and shoot apical meristems.

**Fig. 8.**
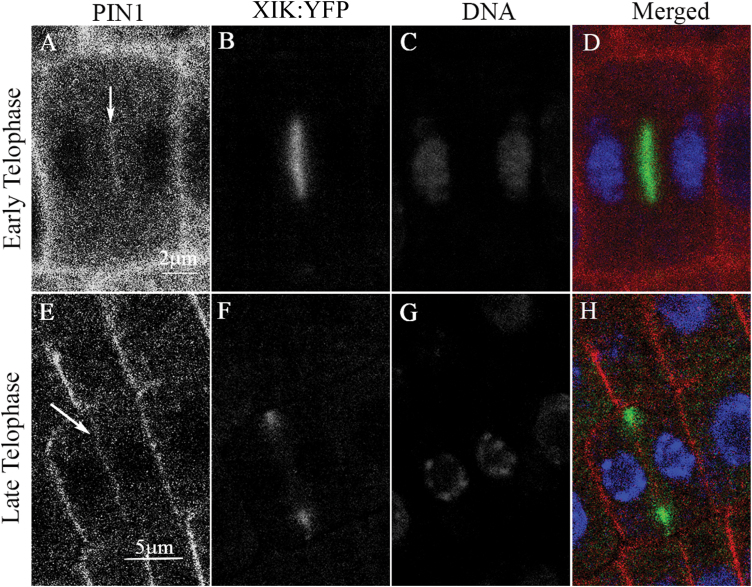
Partial overlap between PIN1 and myosin XI-K:YFP in the cell plates. PIN1 was immunostained in roots of 3KOR plants with PIN1-specific antibody (red); DNA was stained with DAPI (blue). (A–D) A cell during early telophase; the arrow points to PIN1 accumulation in the forming cell plate. (E–H) At late telophase, myosin XI-K:YFP concentrates at the leading edge of the growing cell plate, whereas PIN1 is distributed throughout the cell plate (arrow in ‘E’). Scale bars in (A–D)=2 µm; in (E–H)=5 µm.

As has been previously shown, during cell division, PIN1 is targeted to the forming cell plate (Boutté *et al.*, 2006). To determine possible co-localization of PIN1 and myosin XI-K in cell plates, 3KOR plants were immunostained for PIN1. It was found that in the early telophase, the XI-K:YFP and PIN1 signals largely overlapped in the mid-zone of a dividing cell (Fig. 8A–D). In contrast, during the late telophase, the XI-K:YFP signal was confined primarily to the leading edge of the cell plate, whereas the PIN1 signal was distributed along the entire, newly formed cell plate (Fig. 8E–H). Such separation of the myosin XI-K and PIN1 in late telophase suggests that the mechanism of PIN1 localization along the newly formed plasma membrane and that of myosin targeting to cell plate edges are distinct. Indeed, proper localization of PIN1 to the cell plate can be observed in the 3KO plants (Supplementary Fig. S9), although no statistical analysis was performed due to the limited number of PIN1-expressing dividing cells found in the stele. Taken together, these results suggest that transient myosin XI-K/PIN1 co-localization in the cell plate is not functionally linked to the loss of polar PIN1 distribution in mature stele cells of the 3KO plants.

## Discussion

Significant progress in plant myosin research in the last decade identified myosin functions in polarized and diffuse cell growth, endomembrane transport, and cytoplasmic streaming (Buchnik *et al.*, 2015). At the whole-plant level, myosins were implicated in the growth of aerial plant organs, and morphogenesis of stems, siliques, and flowers, as well as in plant gravitropic responses (Ojangu *et al.*, 2007, 2012; Peremyslov *et al.*, 2008, 2010, 2015; Okamoto *et al.*, 2015). In addition, loss of myosin XI expression hampered the auxin effect on vacuolar morphogenesis (Scheuring *et al.*, 2016). By following auxin responses in plants or BY2 cells treated with 2,3-butanedione monoxime (BDM), a drug that inhibits myosin ATPase activity, it was previously suggested that myosin has an effect on auxin transport (Holweg *et al.*, 2003; Holweg, 2007). Therefore, we were interested in determining if there is a functional connection between myosins and auxin-regulated root development. To this end, we selected Arabidopsis root morphogenesis as a sensitive auxin-responsive model. We also used a myosin triple gene knockout 3KO in which three highly expressed myosin XIs were inactivated, resulting in a developmental phenotype affecting aerial organs (Peremyslov *et al.*, 2010, 2012), whereas the potential effects of myosin loss on root development were not previously investigated. Our study did support a significant role played by myosin XIs in the auxin regulation network and, moreover, it showed that myosins are localized to cell plates in dividing cells and probably function in cell plate growth. Below we briefly discuss four of our most significant findings.

First, we have demonstrated that the 3KO mutant generates significantly more LRs and ARs than the Columbia control, indicating that root morphogenesis is affected by myosin inactivation (Fig. 1; Supplementary Fig. S1). This latter conclusion was validated by the fact that the genetically rescued plant line 3KOR exhibited normal root architecture. Because the initiation of new LRs and ARs is dependent on auxin signaling (Riov *et al.*, 2013; Verstraeten *et al.*, 2014), this result suggests a possible role for myosin in auxin regulation of root morphogenesis. Partial reversal in terms of excessive LR production by 3KO plants upon exogenous auxin treatment further strengthened this possibility (Supplementary Fig. S2). Our data do not provide a ready explanation for why elimination of myosins results in increased rather than reduced LR and AR formation. Current understanding is that the priming of pericycle cells for LR formation occurs in the root transition domain between the meristem and the elongation zone (De Smet *et al.*, 2007). It was also found that the auxin minimum above the meristem zone defines a developmental window for founder cell specification and LR initiation (Dubrovsky *et al.*, 2011). It was further shown that programmed cell death involving the distal edge of the root cap in the transition zone results in a release of auxin signals that affect LR formation (Xuan *et al.*, 2016). Thus, the changed pattern of auxin accumulation at the transition zone in 3KO plants might affect LR induction. Our findings are also in apparent conflict with an earlier work showing that the auxin transport inhibitor NPA reduces LR formation (Casimiro *et al.*, 2001). However, recent work using the natural auxin efflux inhibitor *cis*-cinnamic acid (c-CA) reported increased LR and AR formation in the presence of c-CA (Steenackers *et al.*, 2017). Evidently, follow-up research is needed to pin down the mechanistic connections between inactivation of myosins and changes in root architecture.

Secondly, we found that although the shape of the primary root appeared normal in the 3KO mutant, the morphology of the stele cells was affected: more cells showed an irregular shape and oblique cell walls in contrast to predominantly uniform, rectangular cells in the control plants (Fig. 2). These morphological abnormalities, however, did not affect all stele cells in the 3KO line. In general, lack of more severe cell shape defects could be explained by expression of the other myosin XIs in the stele (such as XI-I, XI-H, and XI-F; Duan and Tominaga, 2018), which could partially compensate for elimination of myosins XI-1, XI-2, and XI-K. Functional redundancy among myosin XIs typically results in a gradual increase in phenotype severity in response to progressive inactivation of the myosin XIs (Prokhnevsky *et al.*, 2008). It was further shown that the stele cell abnormalities in 3KO roots correlated with the reduced auxin response levels in root meristem and a shorter and steeper basipetal auxin response gradient indicative of the defects in polar auxin transport (Fig. 3). This assumption was supported by using the auxin transport inhibitor NPA, which affected the auxin gradient in the roots of Columbia plants in a manner similar to that observed in the 3KO plants. Moreover, using the GFP-tagged auxin efflux transporter PIN1 that is normally distributed in a polar manner to promote proper auxin gradient formation, we found that PIN1–GFP is depolarized in 3KO root cells (Fig. 4). These findings strongly suggest that myosin elimination disrupts the normal localization pattern of PIN1 (and possibly additional auxin transporters), affects auxin gradient formation, and results in abnormalities in stele cell morphogenesis. This latter possibility is supported by findings showing that cell division can be affected by auxin signaling (Bennett and Scheres, 2010) and that inhibition of auxin transport by NPA resulted in abnormal orientation of cell division in suspension-cultured cells (Petrásek *et al.*, 2002).

Our third intriguing finding has to do with the apparent involvement of myosin XIs in plant cell division. To explore myosin XI-K contributions to stele cell wall formation, we investigated the real-time localization of the functional myosin XI-K:YFP in the roots of 3KOR plants. In the fully differentiated leaf and root epidermal cells, XI-K:YFP localizes to the vesicle-like compartments rapidly moving along F-actin bundles (Peremyslov *et al.*, 2012, 2013). In sharp contrast, in the dividing cells of the root meristem, we observed conspicuous disc- and ring-like structures where XI-K:YFP was concentrated (Fig. 5). A series of co-localization studies in both fixed and live cells demonstrated that in metaphase, myosin XI-K:YFP faintly co-localizes with mitotic spindle MTs, but not with the centrally positioned chromatids (Figs 6, 7). This observation is in agreement with previous reports on the faint localization of actin to the spindle (Liu and Palevitz, 1992). Interestingly, at this stage, bright myosin XI-K:YFP fluorescence was also observed at the cell division zone, which later disappeared, but was restored at late telophase. During anaphase, myosin XI-K:YFP congregated in the mid-zone of the spindle. During telophase progression, XI-K:YFP was further concentrated in the phragmoplast mid-zone where the cell plate was forming. Finally, the ring-like appearance of the myosin XI-K:YFP in late telophase implied myosin association with the leading edge of the growing cell plate (Figs 6, 7).

Combined with observations of a slower rate of cell division and higher rate of tilted phragmoplasts and spindles in the root meristem of 3KO plants, these results clearly suggested that myosin XIs contribute to cytokinesis and growth of the cell plate. It should be mentioned that localization of myosin XI to the cell division apparatus was previously reported by using immunostaining of cultured tobacco cells (Yokota *et al.*, 2009). When this manuscript was under review, myosin XI targeting to the cell plate was documented in a moss model (Sun *et al.*, 2018), indicating that potential myosin function in cell division is evolutionarily conserved. Intriguingly, the presence of myosin XIs in the CDZ and cell plate is also reminiscent of observations on myosin VIII in moss (Wu and Bezanilla, 2014). The potential interactions of myosin XI-K with other CDZ-resident proteins (Rasmussen *et al.*, 2013; Wu and Bezanilla, 2014; Buschmann *et al.*, 2015) are yet to be investigated.

Fourthly, much to our surprise, we found no physical association between myosin XI-K and its MyoB1 and MyoB2 receptors in the growing cell plates of dividing cells. In the mature leaf and root cells or root hairs, nearly the entire pool of myosin XI-K:YFP is bound to these membrane-anchored receptors that are highly expressed throughout the plant (Peremyslov *et al.*, 2012, 2013; Kurth *et al.*, 2017) and contribute to cytoplasmic streaming and to stem morphogenesis (Peremyslov *et al.*, 2015). In stark contrast, neither MyoB1 nor MyoB2 was detected in the dividing cells of the root or shoot meristem, suggesting that a presumable myosin XI-K function in cell plate formation depends on the myosin cargo(s) distinct from MyoB1 and MyoB2 vesicles (Supplementary Figs S7, S8). Although it cannot be excluded that other, less abundant MyoB receptors associate with myosins in the growing cell plate, the conspicuous absence of MyoB1 and MyoB2 in both the root and shoot meristems is of potential functional significance. It seems plausible that cessation of cytoplasmic streaming occurring in mitotic cells is required for proper formation of the mitotic spindle, chromosome segregation, and cell plate formation, as suggested by early work (Mineyuki *et al.*, 1984). Furthermore, the absence of these MyoBs possessing high affinity for myosins in meristems could avoid competition with alternative adaptors specifically involved in myosin function in cell division.

There seem to be at least three hypothetical mechanisms whereby myosin XIs could contribute to polar distribution of auxin responses in Arabidopsis roots. The first involves a role for myosin XI-K in the dividing cells of the root meristem, where myosin activity appears to be involved in proper cell plate growth and positioning. In turn, myosin inactivation could result in aberrant cell shapes and a defective auxin gradient. By analogy with the actomyosin-dependent polar growth of the root hairs (Ketelaar, 2013), myosins could be involved in delivery of the building materials toward and within the forming cell plates. As we propose here, such delivery could involve cargos other than MyoB1 and MyoB2 compartments. A second potential mechanism mediating crosstalk between myosin motility and auxin signaling has to do with the requirement for myosin for the proper subcellular accumulation of the auxin transporters in fully differentiated cells. This mechanism could involve the myosin-driven cytoplasmic streaming that facilitates anterograde and retrograde transport of the auxin transporters to and from the plasma membrane. The fact that PIN1 is present in the plasma membrane in the stele cells of 3KO plants but not in a polar manner suggests that the velocity of membrane trafficking which is compromised in the absence of myosins is critical for PIN1 polar maintenance but not for its delivery to the plasma membrane.

The third possible mechanism could involve specific myosin-dependent pathways mediating targeted recycling of auxin transporters to their proper destinations. There are several lines of evidence suggesting that the co-localization of PIN1 and myosin XI-K at the cell plate is not a prerequisite for the subsequent polar distribution of PIN1: (i) PIN1 is present in the cell plates in the 3KO plants; (ii) polar distribution of PIN1 was restored after BFA washout in the Columbia non-dividing cells, but not in the cells of 3KO plants; and (iii) it was demonstrated that PIN1 is initially distributed to both sides of cell plates (Boutté *et al.*, 2006), suggesting that its polarization is not yet established at this stage. Of note, unlike the transient co-localization with myosin XI-K, the association of PIN proteins with dynamin-related proteins in the cell plate during cytokinesis is required for the proper polarization of these PIN proteins in interphase (Mravec *et al.*, 2011).

In conclusion, the presented data imply that myosin XI-K and probably other accessory myosin XIs contribute significantly to the polar distribution of auxin signaling. The exact molecular mechanisms mediating the interplay between the myosin-driven transport and auxin regulation network is a subject for future research.

## Supplementary data

Supplementary data are available at *JXB* online.

Movie S1. Cell division in the 3KOR plant expressing myosin XI-K:YFP and the RFP–MBD microtubule marker.

Fig. S1. Representative images showing that the 3KO plants produce more lateral roots than Columbia or 3KOR plants upon growth for 5 d in the dark and 4 d in the light (9 d total).

Fig. S2. The increased lateral root (LR) density phenotype of the 3KO plants is rescued in the presence of ectopic IAA.

Fig. S3. DR5 fluorescence accumulation during LR primordium formation in Columbia and 3KO plants..

Fig. S4. Immunostaining with PIN1-specific antibody shows loss of polarization of PIN1 in 3KO plants in contrast to Columbia and 3KOR plants.

Fig. S5. BFA treatments followed with washouts show reversible PIN1:GFP relocation from the BFA compartment and depolarized to polarized distribution in Columbia root tips versus no changes in the depolarized pattern of PIN1:GFP in 3KO plants.

Fig. S6. Tilted phragmoplast and spindles in the dividing root cells of the 3KO plants expressing the red microtubule marker mCherry–MBD.

Fig. S7. Visualization of the MyoB1–GFP, MyoB2–GFP, and myosin XI-K:mCherry in the root apical meristem and in the elongating root hairs.

Fig. S8. Visualization of the MyoB1–GFP and myosin XI-K:mCherry in the shoot apical meristem and in the petiole epidermis.

Fig. S9. PIN1:GFP can be observed in the 3KO root cell plate suggesting that its targeting to this site is independent of myosins XI-K, XI-1 and XI-2.

Supplementary Figures

Supplementary Movie
