# Supplementary material for: Myosin XI-K is involved in root organogenesis, polar auxin transport, and cell division
Source: J Exp Bot. 2018 Mar 22;69(12):2869–81. doi: 10.1093/jxb/ery112 (PMC5972647; doi:10.1093/jxb/ery112)
Supplement: Supplementary Figures [file ery112_suppl_supplementary_figures.pdf]

Myosin XI-K is involved in root organogenesis, polar auxin transport and cell division

Mohamad Abu-Abied[1]*, Eduard Belausov[1]*, Sapir Hagay[1], Valera Peremyslov[2], Valerian Dolja[2] and Einat Sadot[1#]

[1] The Institute of Plant Sciences, The Volcani Center, ARO, HaMaccabim Road, Rishon LeZion, 7528809, Israel.

[2]Department of Botany and Plant Pathology, Oregon State University, Cordley Hall 2082, Corvallis, OR 97331, USA

Supplemental figures

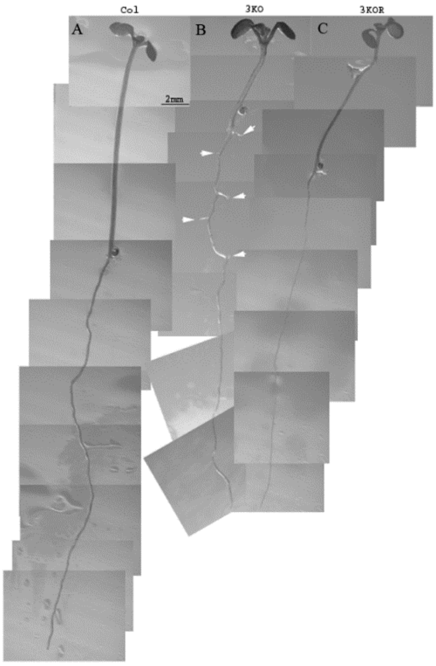

Fig. S1. Representative images showing that the 3KO plants produce more lateral roots than Columbia or 3KOR plants upon growth for 5d in the dark and 4d in the light (9d total).

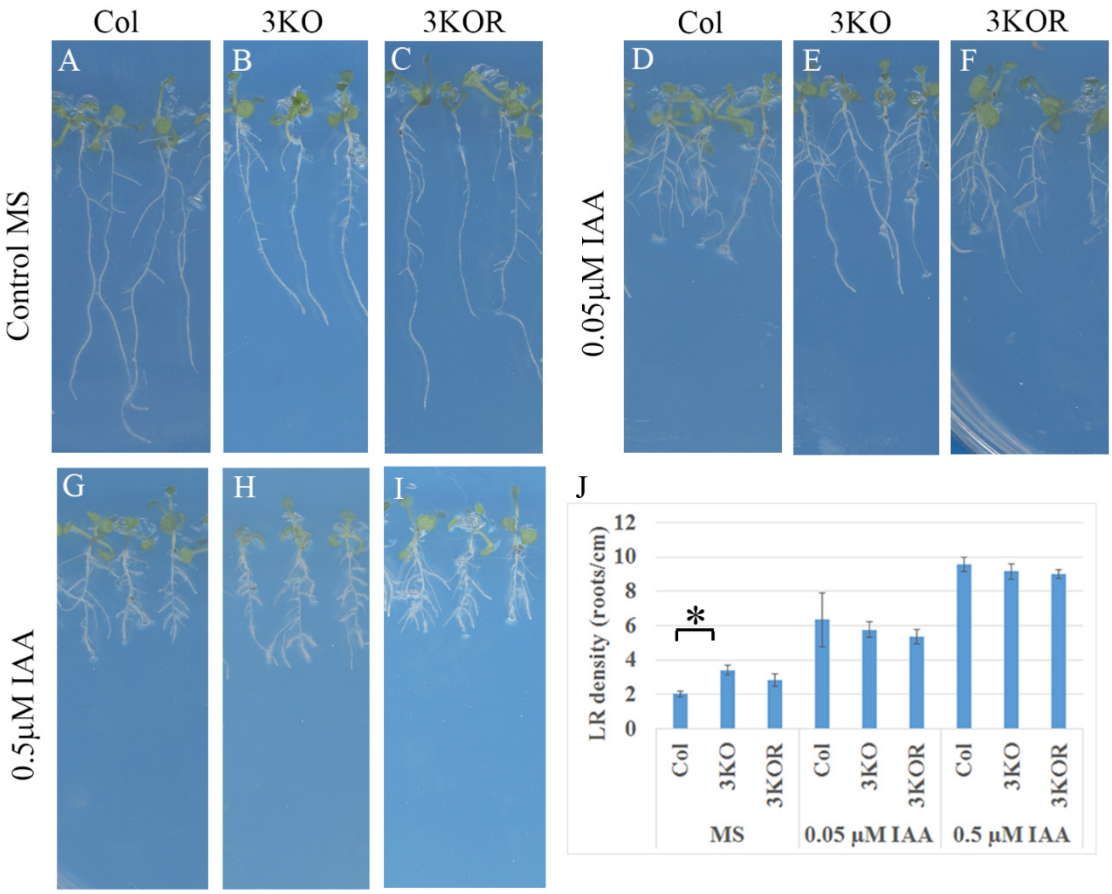

Fig. S2. The increased lateral root (LR) density phenotype of the 3KO plants is rescued in the presence of ectopic IAA. (A-I) Representative plant images for the control and treated plants as indicated. (J) The LR density in the absence (MS) or presence of IAA as indicated. Data were analyzed by Scheffe analysis. Error bars show standard error. The experiment was replicated 3 times, 10 seedlings of each plant line in each treatment. Asterisk marks statistically significantly difference between Columbia and 3KO lines ($p<0.05$).

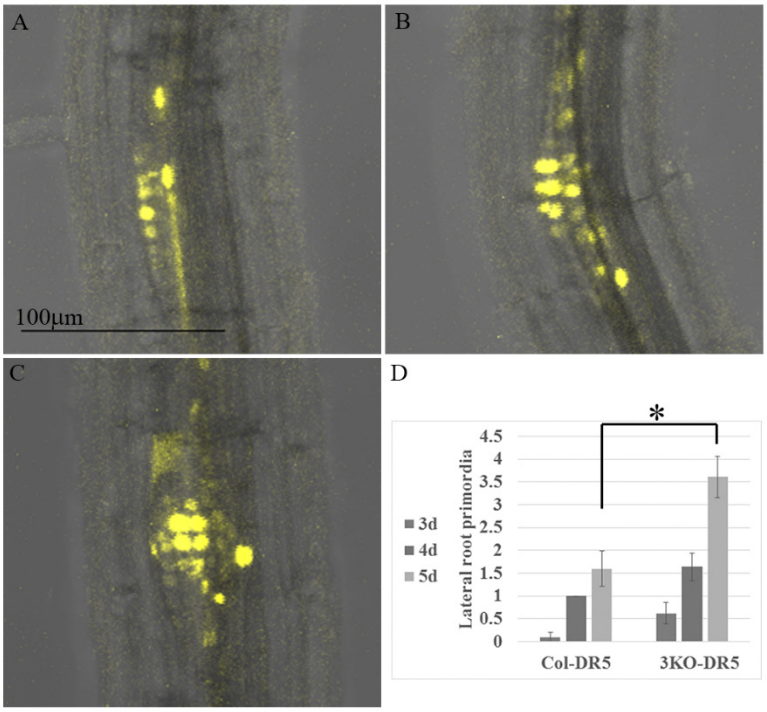

Fig S3. DR5 fluorescence accumulation during LR primordium formation in Columbia and 3KO plants. (A-C) Auxin response in the lateral root (LR) primordium exhibited as accumulation DR5:venus fluorescence in 3KO plant imaged 5d post germination. (A) Early stage of LR primordium showing DR5-YFP in the pericycle. (B, C) Later in the process, additional layers of dividing cells are visible accumulating DR5 signal. (D) Quantitative analysis of the number of such early primordium (1-4 layers of fluorescent cells starting from the pericycle) observed in the two plant lines.

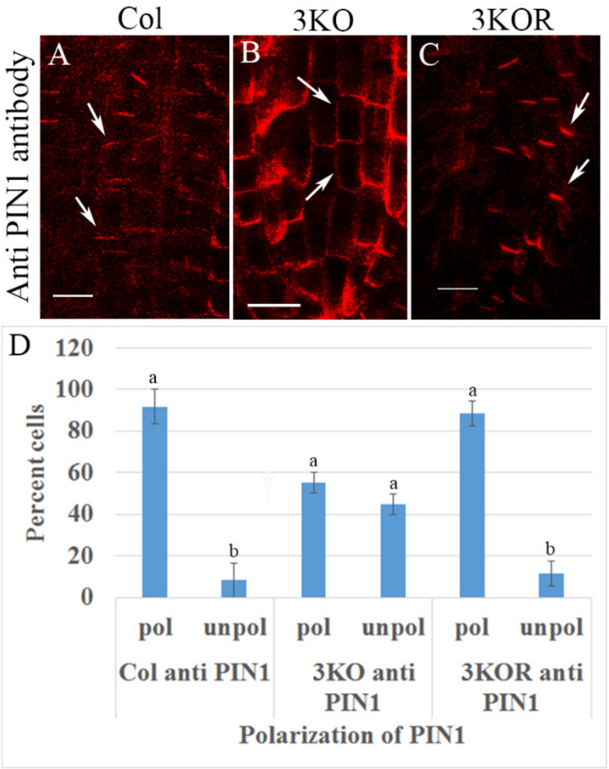

Fig. S4. Immunostaining with PIN1-specific antibody shows loss of polarization of PIN1 in 3KO plants in contrast to Columbia and 3KOR plants. Ten roots of each Columbia and 3KOR plants, and 20 roots of 3KO plants were examined. Columns marked with 'a' show no statistically significant differences, whereas each of 'b' columns is significantly different from each 'a' column. Error bars show standard error.

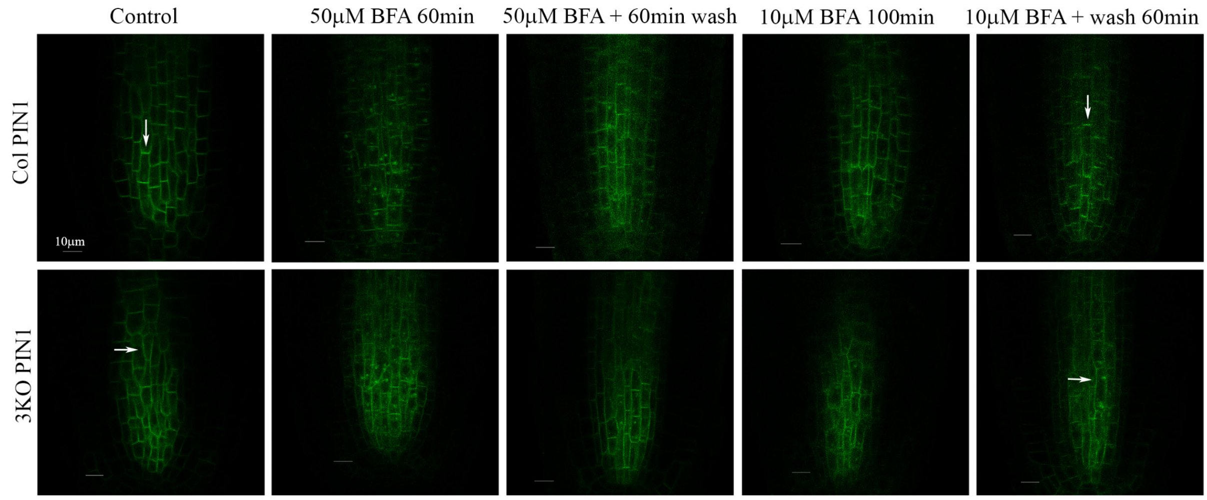

Fig. S5. BFA treatments followed with washouts show reversible PIN1:GFP relocation from BFA compartment and depolarization to polarized distribution in Columbia root tips versus no changes in depolarized pattern of PIN1:GFP in 3KO plants. The BFA concentrations and treatment times are as indicated. Bars, 10μm.

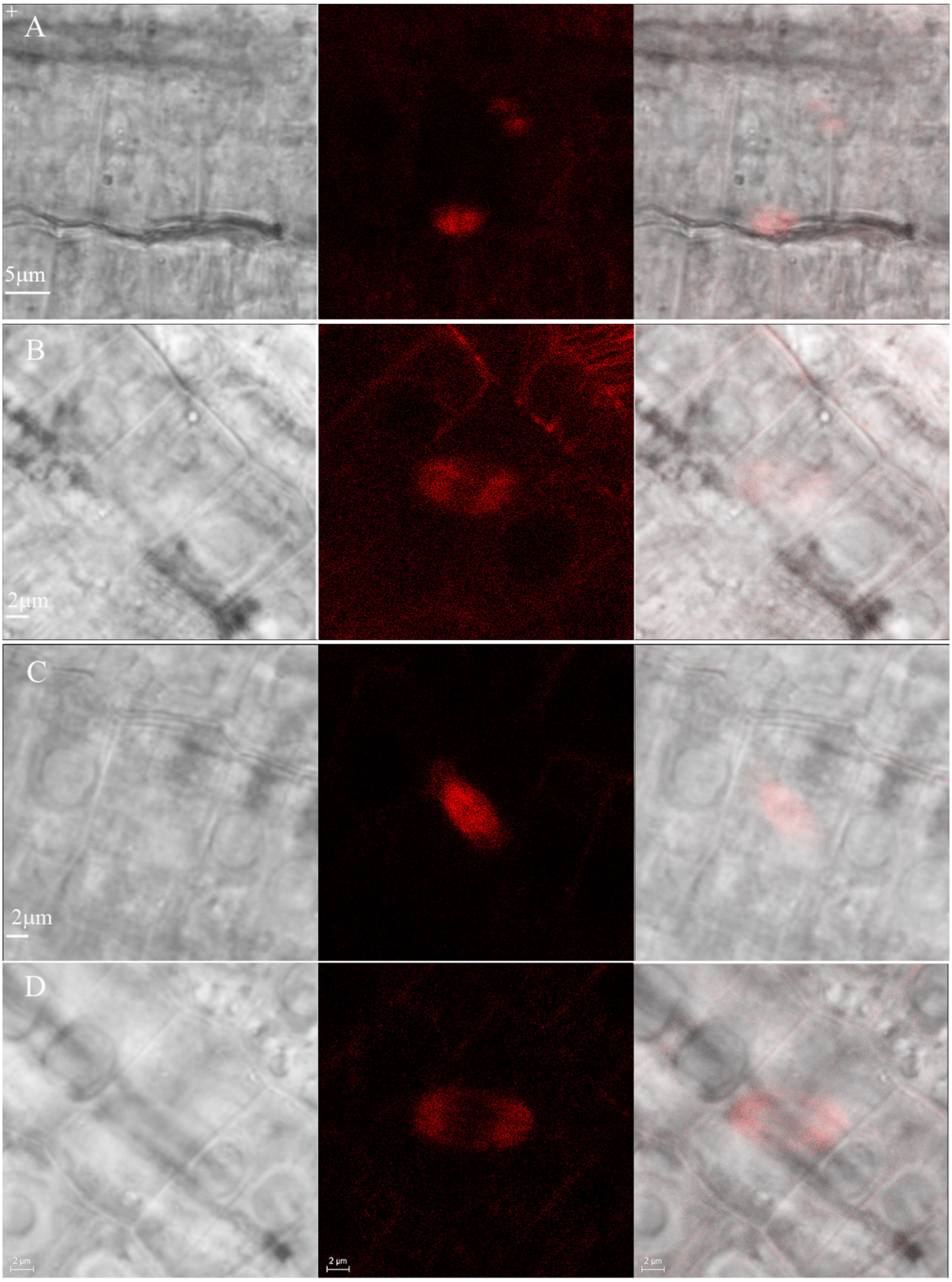

Fig. S6. Tilted phragmoplast (A) and spindles (B-D) in the dividing root cells of the 3KO plants expressing the red microtubule marker mCherry-MBD.

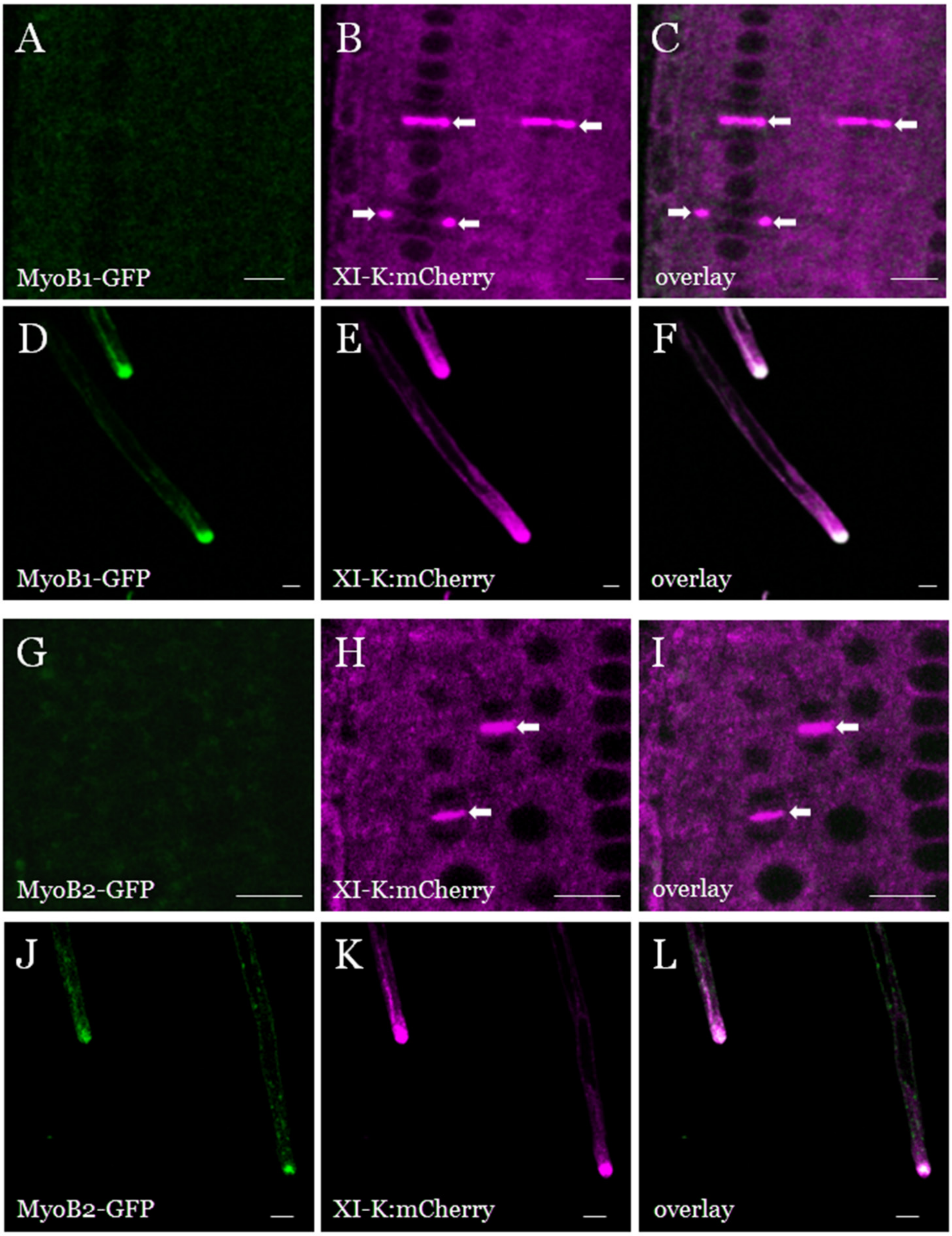

Fig. S7. Visualization of the MyoB1-GFP, MyoB2-GFP and myosin XI-K:mCherry in the root apical meristem (A-C; G-I) and in the elongating root hairs (D-F; J-L). (A) No expression of the MyoB1-GFP is detectable in root meristem. (B and C) The myosin XI-K:mCherry localizes to the plates of diving meristematic cells (arrows). (D-E) The MyoB1-GFP and the myosin XI-K:mCherry co-localize to the tips of growing root hairs. (G) No expression of the MyoB2-GFP

is detectable in root meristem. (H and I) The myosin XI-K:mCherry localizes to the plates of diving meristematic cells (arrows). (J-L) The MyoB2-GFP and the myosin XI-K:mCherry co-localize to the tips of growing root hairs. Bars, 10 μm.

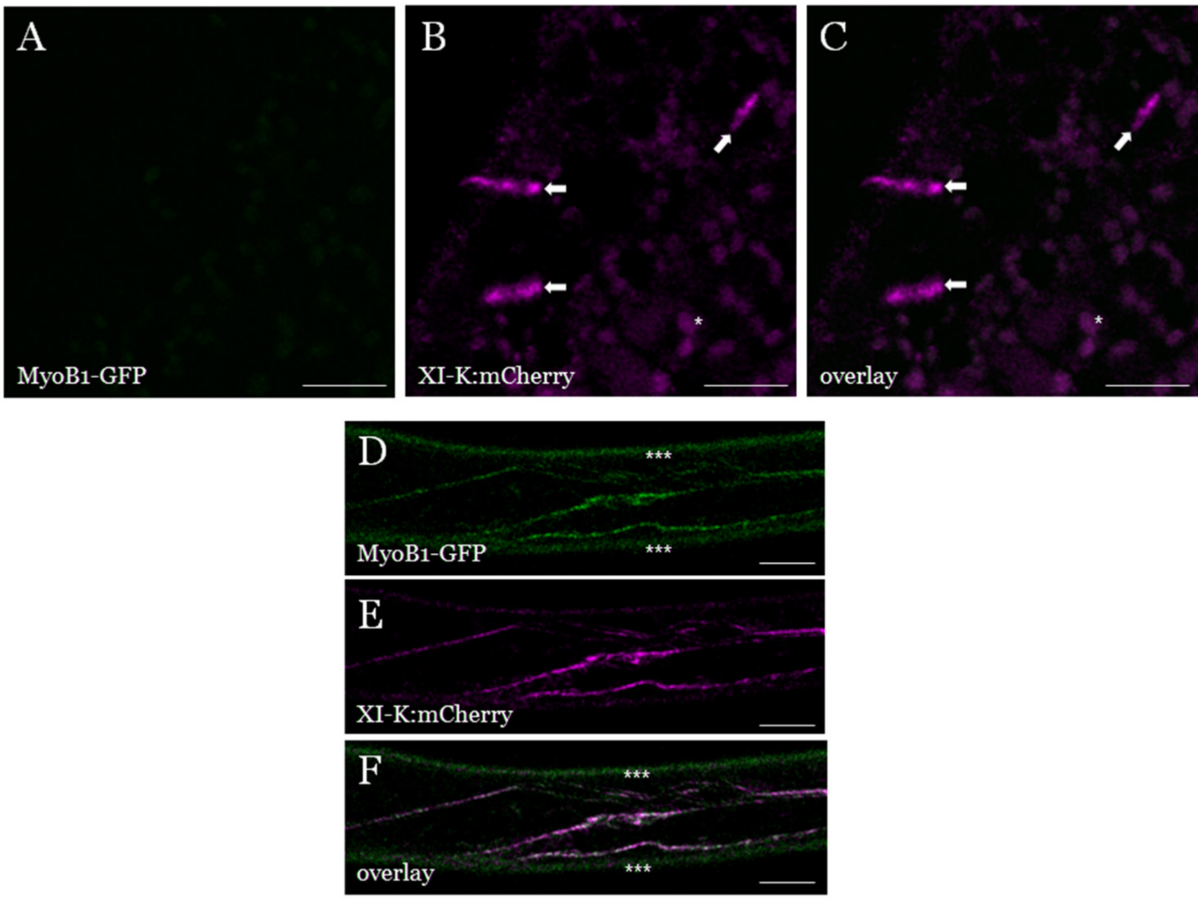

Fig. S8. Visualization of the MyoB1-GFP and myosin XI-K:mCherry in the shoot apical meristem (A-C) and in the petiole epidermis (D-F). (A) No expression of the MyoB1-GFP is detectable in apical meristem. (B and C) The myosin XI-K:mCherry localizes to the plates of diving meristematic cells (arrows). Asterisk marks one of the autofluorescent chloroplasts visible throughout the image. (D) Expression of MyoB1-GFP visualizes typical linear vesicle-like arrays within the cells; asterisks mark autofluorescent cell walls (D). (E) Expression of myosin XI-K:mCherry shows similar linear arrays that co-localize with MyoB1-GFP (F). Bars, 10 μm.

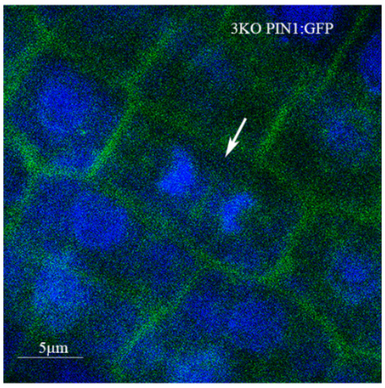

Fig. S9. PIN1:GFP can be observed in the 3KO root cell plate suggesting that its targeting to this site is independent of myosins XI-K, XI-1 and XI-2.
